# On the Necessity of a Customized Knee Spacer in Peri-Prosthetic Joint Infection Treatment: 3D Numerical Simulation Results

**DOI:** 10.3390/jpm11101039

**Published:** 2021-10-17

**Authors:** Marco Balato, Carlo Petrarca, Vincenzo de Matteo, Marco Lenzi, Enrico Festa, Andrea Sellitto, Jessica Campi, Mauro Zarrelli, Giovanni Balato

**Affiliations:** 1 Department of Electrical Engineering and Information Technologies, University of Naples “Federico II”, 80125 Napoli, Italy; carlo.petrarca@unina.it; 2 Department of Public Health, University of Naples “Federico II”, 80131 Napoli, Italy; vincenzo.dematteo@studenti.unina.it (V.d.M.); marco.lenzi@studenti.unina.it (M.L.); enrico.festa@studenti.unina.it (E.F.); giovanni.balato@unina.it (G.B.); 3 Department of Engineering, University of Campania “Luigi Vanvitelli”, 81031 Aversa, Italy; andrea.sellitto@unicampania.it (A.S.); jessica.campi@studenti.unicampania.it (J.C.); 4 National Research Council of Italy (CNR), Institute for Polymers, Composite and Biomedical Materials (IPCB), 80055 Portici, Italy; mauro.zarrelli@cnr.it

**Keywords:** custom made knee spacer, peri-prosthetic infection, two stage knee revision, virtual surgery

## Abstract

Peri-prosthetic joint infections (PJIs) dramatically affect human health, as they are associated with high morbidity and mortality rates. Two-stage revision arthroplasty is currently the gold standard treatment for PJI and consists of infected implant removal, an accurate debridement, and placement of antimicrobial impregnated poly-methyl-metha-acrylate (PMMA) spacer. The use of antibiotic-loaded PMMA (ALPMMA) spacers have showed a success rate that ranges from 85% to 100%. ALPMMA spacers, currently available on the market, demonstrate a series of disadvantages, closely linked to a low propensity to customize, seen as the ability to adapt to the patients’ anatomical characteristics, with consequential increase of surgical complexity, surgery duration, and post-operative complications. Conventionally, ALPMMA spacers are available only in three or four standard sizes, with the impossibility of guaranteeing the perfect matching of ALPMMA spacers with residual bone (no further bone loss) and gap filling. In this paper, a 3D model of an ALPMMA spacer is introduced to evaluate the cause- effect link between the geometric characteristics and the correlated clinical improvements. The result is a multivariable-oriented design able to effectively manage the size, alignment, stability, and the patients’ anatomical matching. The preliminary numerical results, obtained by using an “ad hoc” 3D virtual planning simulator, clearly point out that to restore the joint line, the mechanical and rotational alignment and the surgeon’s control on the thicknesses (distal and posterior thicknesses) of the ALPMMA spacer is mandatory. The numerical simulations campaign involved nineteen patients grouped in three different scenarios (Case N° 1, Case N° 2 and Case N° 3) whose 3D bone models were obtained through an appropriate data management strategy. Each scenario is characterized by a different incidence rate. In particular, the observed rates of occurrence are, respectively, equal to 17% (Case N° 1), 74% (Case N° 2), and 10% (Case N° 3).

## 1. Introduction

Joint arthroplasty (JA) surgery has increased in the last few years with a further increase by 284% for primary total hip arthroplasty (THA) and 401% for total knee arthroplasty (TKA) has been estimated to be recorded in the next two decades [1,2,3,4,5]. Despite the low complications rate after joint replacement, several patients will require additional surgery due to aseptic or septic failures [6,7,8]. Aseptic loosening complications have been reported up to 28% of the total JAs, including peri-prosthetic fracture, prosthetic loosening, material wear, implant malposition, or instability [8].

Peri-prosthetic joint infection (PJI) represents a dramatic post-operative complication that occurs in a percentage of patients that ranges from 1 to 2%. However, the incidence rate is steadily increasing with an estimated rise projection by 137% between 2005 and 2030 for hip revision in the US and a national total cost of $753.4 millions of dollars [8,9,10,11,12,13]. Similarly, an increase of the national total hospital cost for periprosthetic knee infection TKA up to $1.1 billion US dollars annually has been described. Two-stage revision arthroplasty is currently the gold standard treatment for PJI that consists in infected implant removal, an accurate debridement, and placement of an antimicrobial impregnated poly-methyl-metha-acrylate (PMMA) spacer. The use of antibiotic-loaded PMMA (ALPMMA) spacers have showed a success rate that ranges from 85% to 100% [14,15,16,17]. Differently from static spacers, ALPMMA articulating spacers are designed to: (i) preserve anatomical structures, reduce arthrofibrosis (scar-tissue formation); (ii) maintain the joint range of motion thus increase the patient’s quality of life during the interim period between the first and second surgical procedure; and finally (iii) allow an easier and more effective re-implantation of the definitive prosthesis during the second stage [18,19,20,21,22,23,24,25]. However, and despite all of the advantages that are correlated to the geometric shape of the available articulating spacers, they are not highly customizable with consequential decrease of the surgeon’s possibility to deal specific anatomical conditions such as bone defects. Indeed, ALPMMA spacers are available only in three or four standard sizes with the impossibility to: (i) guarantee perfect matching with residual bony anatomy; (ii) restore the frontal and rotational deformities; and (iii) provide an appropriate gap filling and soft tissue tensioning. The above statements clearly highlight the necessity for a new concept of an articulating spacer, based on the customization process, as a powerful tool to guarantee the perfect matching of spacers with residual bone (without causing further bone loss), gap filling, and axis correction, so that postoperative motion is preserved as the collateral ligaments and patellar tendon’s length are maintained. A careful analysis of the scientific literature has shown that the concept of customization of ALPMMA spacers is not an innovative issue, as it was introduced for the first time several years ago. Nevertheless, presently, there is still no consensus on femoral spacer’s morphological characteristics since no investigations have been conducted for the introduction of guidelines on spacers design to combine the geometric characteristics with the correlated clinical improvements. Therefore, the real advantages of customization have not been pointed out yet. Moreover, the pros and cons of a systemic use in orthopedic practice are still mostly unknown. The first step to seriously address this issue, with the aim of advancing scientific knowledge, is the introduction of a 3D model of the ALPMMA spacer with many degrees of freedom. In the authors’ opinion, this is the only way to study the randomness link (cause-effect link) in detail. For this aim, an innovative Virtual 3D planning simulator has been developed. Our preliminary numerical results highlight that, by acting on the distal and posterior thicknesses, it is possible to restore both the joint line and the mechanical and rotational alignment. As shown in the following, the cases study involves nineteen patients, whose 3D bone characteristics, obtained by a suitable data management process, allow the definition of three different scenarios.

## 2. Materials and Methods

### 2.1. ALPMMA Spacer 3D Modeling

**Figure 1:**
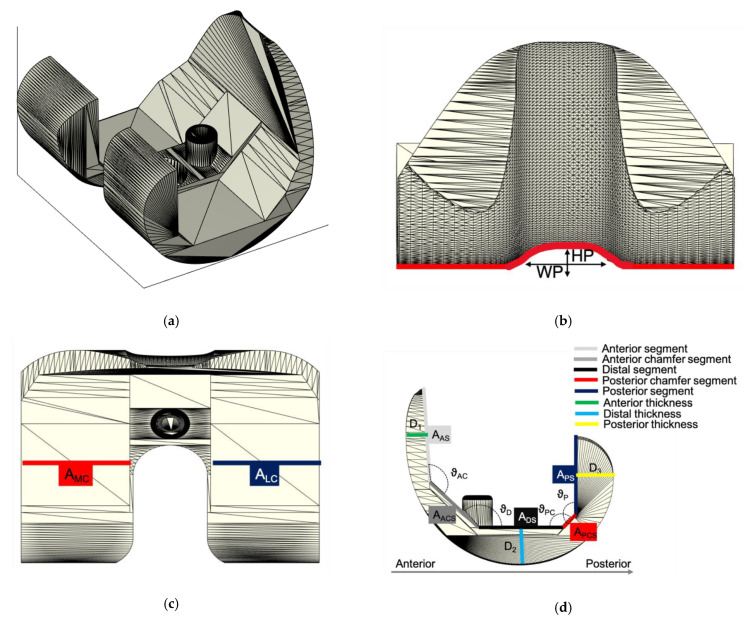
3D model of the ALPMMA spacer: (**a**) 3D view of the proposed ALPMMA femoral component; (**b**) frontal view of the proposed ALPMMA femoral component; (**c**) top view of the proposed ALPMMA femoral component; and (**d**) lateral view of the proposed ALPMMA femoral component.

**Figure 2:**
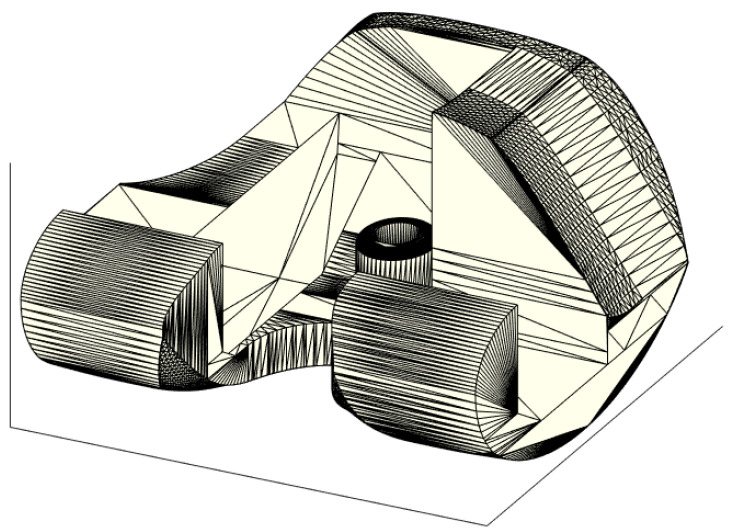
3D view of the proposed augmented ALPMMA femoral component.

The 3D model of the ALPMMA spacer (Figure 1) has been designed using Simulink 3D Animation Matlab Toolbox (The MathWorks Inc. Apple Hill Drive, Natick, MA, USA). It consists of femoral component imitating the geometry of a standard primary total knee prosthesis. To evaluate the cause-and-effect link between the geometric characteristic of the ALPMMA spacer and surgery failures, a multivariable-oriented design was implemented. The result is an ALPMMA spacer with many degrees of freedom with respect to geometric variables acting to effectively manage the size, the alignment, the stability, and the anatomical matching. Prosthetic femoral condyles were shaped on both sides to effectively reduce pressure on the soft tissue and on the anterior patella. Moreover, to improve flexion and extension movement and to reduce the risk of postoperative dislocation and joint instability, it is possible to customize the shape of the anterior condyles and the engaging cam-post mechanism (between femoral and tibial component, red curve of Figure 1b) by acting on the parameters HP and WP that affect its thickness and amplitude, respectively. These parameters will also have effects on the trochlear groove or cam, that will help the patellar enter the carriage movement early. Additionally, the proposed 3D model allows one to carefully plan the encumbrance of the femoral component both in antero-posterior (A–P) and medio-lateral (M–L) size. The objective is to obtain an ALPMMA spacer with an arbitrary ratio between the A-P and M-L size as a powerful tool to correct axial deformities, to guarantee a proper fixation, and to manage bone defects properly. From Figure 1c,d, it is evident how the footprint of the ALPMMA femoral spacer can be modified by editing the thickness (D_1_, D_2,_ and D_3_), the augmentation amplitude of the two femoral internal condyles (A_MC_ and A_LC_), and the amplitude of cutting segments (A_AS_, A_ACS_, A_DS_, A_PCS,_ and A_PS_) and angles (ϑ_AC_, ϑ_D_, ϑ_PC,_ and ϑ_P_). Lastly, an augmented ALPMMA femoral spacer was expected to adapt the spacer to severe bone defects and to correct them. In Figure 2, an example of an asymmetric augmented ALPMMA femoral spacer was reported.

### 2.2. Patient Data Management “3D Bone Model”

**Figure 3:**
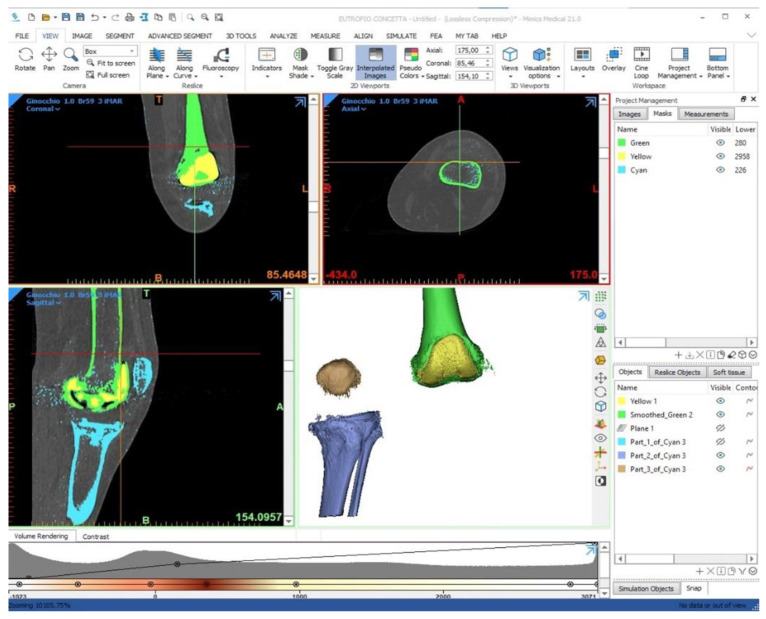
3D view of the proposed augmented ALPMMA femoral component.

The 3D model prototyping process starts from the acquisition of a dataset, generally from a computed tomography (CT) scan of the involved bone segment. It is important to include the “proximal” and “distal” joint to the knee, specifically the hip and the ankle joints, thus lead to identify the lower limb mechanical and anatomical axes and eventually to produce a spacer with an appropriate three-dimensional alignment. In case of severe metal artifacts (presence of multiple hypodense striae) due to the presence of prosthetic implants, a specific MAR (metal artifact reduction) algorithm combined with the dual-energy CT (DECT) is used to produce a good quality image [26]. The MAR techniques are based on a method called “inpainting” that identifies the projection data corrupted due to the presence of metal hardware and replaces them with data mediated or interpolated by the surrounding detector elements, to reduce both the beam hardening, as the effects of photon starvation and scattering. Once an adequate dataset has been obtained, the segmentation phase is carried out using Mimics Innovation Suite© software (MATERIALISE Technology 15, Leuven, Belgium), thus isolating the anatomical part of interest and prosthetic material from the radiological images. The most used techniques are region-based segmentation, which identifies the regions that satisfy a given homogeneity criterion, and the edge-based segmentation, which searches for margins between regions with different characteristics (Figure 3). After segmentation, 3DMatic © software (MATERIALISE Technology 15, Leuven, Belgium), allows various processing on the 3D model, such as checking for errors in the mesh, closing small holes, elimination of external fragments, sanding of surfaces, and analysis of wall thickness.

**Figure 4:**
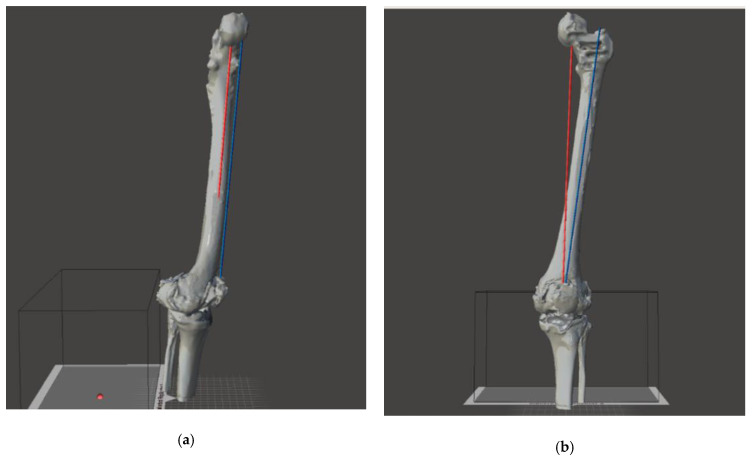
Frontal (**a**) and lateral (**b**) view of mechanical (red) and anatomical (blue) axis drawing.

Using 3D manipulation programs, such as Meshmixer^®^ (Autodesk, Inc. 111 McInnis Parkway San Rafael, CA, USA) or Blender^®^ (Blender Foundation Buikslotermeerplein 161, Amsterdam The Netherlands), an anonymizing univocal sequential code was firstly impressed on the mesh, then the anatomical and mechanical axes were described on the femur and the tibia of the virtual limb (see Figure 4). Finally, the meshes were exported as a “.stl” file format. This process aims to produce a virtual model that can be imported in Matlab environment with which a 3D virtual planning can be carried out. The use of common manipulation programs, such as Meshmixer and Blender, is therefore only limited to obtain the patients’ 3D bone model in an “stl” file format, since “stl” is the file format used in the newly developed 3D planning simulator.

### 2.3. Patient Characteristics

For the present ex-vivo investigation, diagnostic records of nineteen consecutive patients (11 males and 8 females with a mean age of 56 years) who were candidate to the second stage knee revision were recruited between April and September 2020. All of the data were acquired after obtaining patient’s permission and informed consent. All patients were affected by peri-prosthetic knee infection associated with mild to moderate femoral bone loss [27]. International consensus meeting on PJI diagnostic criteria based on clinical and laboratory investigations were used to identify the presence of joint infection [28].

### 2.4. Virtual Planning

**Figure 5:**
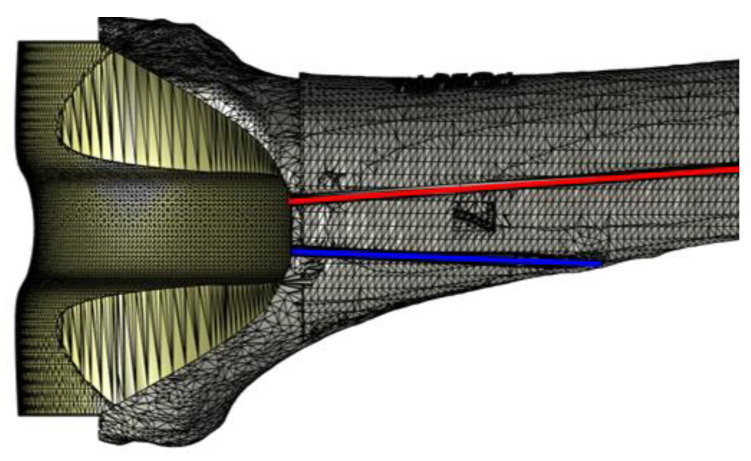
Frontal view of a virtually reconstructed femur, with a representation of the mechanical (red line) and anatomical (blue line) axis.

The virtual planning aims to design a customized revision surgery through an accurate evaluation of the patient’s condition. All virtual models were imported in the Matlab environment. A multidisciplinary team has carried out the virtual planning using the previously designed spacer. All of the morphologic variables were used to adapt the virtual spacer to patients’ anatomy, applying all the needed corrections. The femoral component was firstly molded to produce the correct size, using the tools provided by the program. In a second phase the femoral component was “implanted” perpendicular to the mechanical axis and at distance from the adductor’s notch of 40–45 mm [29]. The implant rotation was obtained according to the trans-epicondylar line on the transverse plane [30]. A custom augmentation was applied at the distal and posterior side, if needed. The study of a lower limb alignment and joint prosthesis orientation represents a key point of the preoperative planning, and it consists of the identification of mechanical and anatomical axis of the femur. Femoral anatomical axis is the axis between two points of the femoral shaft. There is no agreement in the literature on which items should be referenced. According to Moreland’s method [31] the first point is located by bisecting the proximal-to-distal length of the femur (as defined by a line from the superior aspect of the femoral head to the distal part of the medial condyle) and the mid-shaft medial-to-lateral width of the femur. The second point is located 10 cm above the surface of the knee joint midway between the medial and lateral surfaces (blue line of Figure 5). The femoral mechanical axis (FMA) is the axis between the center of the femoral head identified using Mose circles [32] and the center of the femoral component trochlear notch. The mechanical axis could be identified as a line that links together the center of the femoral head to the center of the knee in case of the femoral mechanical axis (red line of Figure 5) and connects the femoral head to the center of the ankle in case of the lower limb mechanical axis. These two fundamental axes are reported on the surface of our virtual model and provide the evaluation of the coronal alignment of the femoral prosthesis that should be perpendicular to the mechanical axe. In addition, in pre-operative planning, other information should be considered to: (i) allow surgeons to overcome all issues in revision surgery; (ii) obtain a component alignment restoration on frontal and axial plane; and (iii) preserve joint line. Alignment restoration represents a challenge to face bone defects that involve femoral condyles thus generating a deviation of the lower limb axes, (towards the external side -> varus deviation; towards the internal side -> valgus deviation). In these scenarios, a personalized spacer femoral component should provide the use of an asymmetrical augmentation at the deficient side, which could restore a correct mechanical axis. Indeed, when the bone defect involves posterior condyles, a rotational axial malalignment should occur with an impact on patella tracking and a correct flex-extension movement. A rotational correction could be achieved with an asymmetrical posterior augmentation. The amount of rotation needed to be corrected must be planned on a CT scan, drawing two lines: one tangent to the posterior condyles and one connecting the prominence of the lateral epicondyle to the sulcus of the medial epicondyle (surgical TEA). The angle between these two lines is the posterior condylar angle (PCA) [30]. Furthermore, the joint line restoration represents another important goal to achieve in revision arthroplasty. The joint line in TKA is defined as the line through the distal aspect of the femoral condyles and influences the patellar height and the knee range of motion. This statement derives from the fact that this compartment is defined from anelastic structures: anteriorly the extensor mechanism, medially, and laterally the collateral ligaments, and ad posteriorly the posterior cruciate ligament and the capsule. Those structures must be tensioned in a proper way to ensure the joint stability and movement. In many cases, the epiphysis of the bones (the segment of the bones that take part into the articulation) present a defect (a bone loss) which must be restored to “fill in the gap” of the articular space. Regarding a femoral defect, a symmetrical augmentation of the “distal” part is appropriate to restore the femoral length. In selected case the joint line could be moved from the original position with the purpose to adjust its position relatively to the patella position.

## 3. Results

### 3.1. CASE N° 1: “Joint Line Restoration (Femoral Distal Symmetrical Augmentation)”

**Figure 6:**
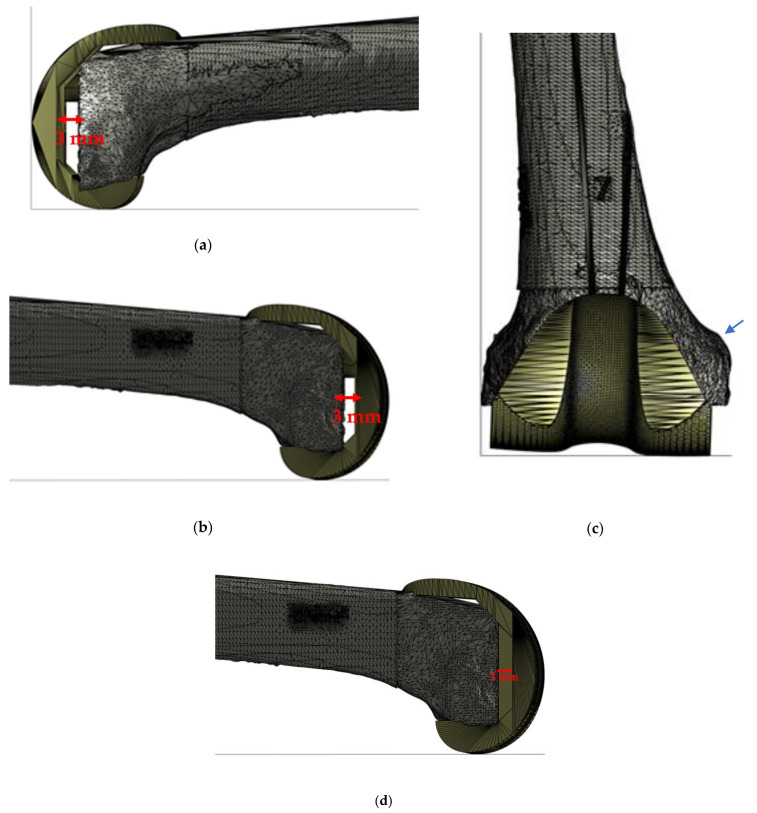
Virtual planning results (Case N° 1): (**a**) and (**b**) lateral view of the right knee with the evidence of bone defect of about 3 mm involving medial and lateral condyle; (**c**) frontal view of the right knee; and (**d**) customization result with an increase of medial and lateral distal thickness.

The lateral view shown in Figure 6a,b demonstrates the need of a “gap filling” from the distal part of the femur to the expected joint line. Generally, the joint line is expected to be placed 40–45 mm from the notch above the adductor’s tubercle Figure 6c (blue arrow). This case highlights the need of a (3 mm) symmetrical augmentation of the femoral component, as illustrated in Figure 6c. In our case series the proximalization of joint line occurred in the 16% (3/19) of patients included in the study.

### 3.2. CASE N° 2: “Alignment Defect Restoration (Femoral Distal Asymmetrical Augmentation)”

**Figure 7:**
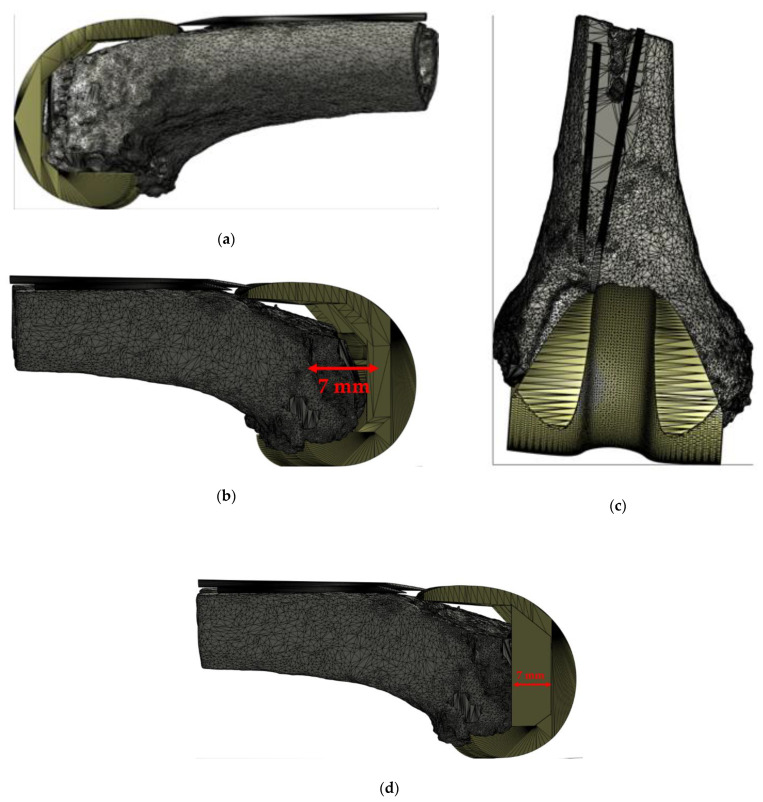
Virtual planning results (Case N° 2): (**a**) and (**b**), lateral view of the left femur with an evidence of bone loss exclusively on medial condyle (**b**); (**c**) correct position of the spacer in the frontal plane; and (**d**) customization result with an increase of medial distal thickness.

In this case the patient presented a bone defect involving only medial femoral condyle (Figure 7), thus generating a varus deformity. The revision strategy includes the use of an asymmetrical augment of (7 mm) on the medial side, restoring the ideal lower limb alignment. In our case series, alignment defect restoration occurred in the 74% (14/19) of patients included in the study.

### 3.3. CASE N° 3: “Rotational Defect Restoration (Femoral Posterior Asymmetrical Augmentation)”

**Figure 8:**
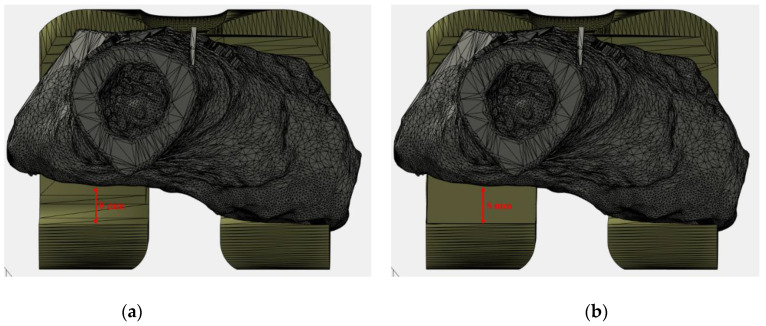
Virtual planning results (Case N° 3): (**a**) posterior view with an evidence of severe bone loss exclusively on posterior-lateral condyle; (**b**) customization result with an increase of posterior-lateral thickness.

The “posterior view” of the Figure 8 shows a posterior-lateral defect. This category of defects produces an improper inward rotation. To restore this defect a posterior asymmetrical augmentation is needed on the lateral side (in this case an 8 mm augmentation was used). The amount of rotation to be restored must be planned pre-operatively. In our case series, rotation defect restoration occurred in the 10% (2/19) of patients included in the study.

## 4. Discussion

Digital orthopedics, based on computer-assisted surgery, represents a new trend in the orthopedic field [33,34,35]. This new kind of approach aims to drastically transform the planning and execution of orthopedic surgeries through the innovative use of ideation, modeling, and simulation technology. Digital orthopedics represents a combination of information technologies and clinical procedures, involving different disciplinary areas such as medicine, computer science, artificial intelligence, robotics, mechanical engineering, and other related areas. The result is patient-oriented resources as a powerful tool to help clinicians improve and manage the entire surgical procedure, resulting in enhanced patients’ functional and clinical outcomes and their quality of life. Moreover, this new frontier can be also used, as virtual instruments, for training young physicians to improve their skills. The goal is to establish a virtuous circle between the knowledge, seen as technical abilities, and the surgical procedures. The general application of digital technology in the orthopedic field involves the three surgical procedure times and/or phases: preoperative phase, intraoperative phase and postoperative phase. Digital preoperative phase is devoted to 3D reconstruction of the patients’ anatomic landmarks via image management processes, including acquisition [36,37,38,39,40], enhancement [41,42,43,44,45], and segmentation techniques and/or procedures [46,47,48,49,50,51]. The purpose is a digital platform “Virtual 3D Planning Simulator (V3DPS)” useful for understanding patient anatomy and planning out a strategy of reconstruction.

An evolution of V3DPS involves the application of 3D printing that may increase the efficacy and reduce surgical morbidity through a process defined as “Simulated Surgical System”. Through a “rapid prototyping” or “additive manufacturing” process, the surgeon goes way beyond utilizing implants which are manufactured based on individual 3D-data (CT, MRI); he can plan all the phases of the surgical procedure and face complex cases such as the management of periprosthetic joint infection. [52].

Periprosthetic joint infection (PJI) is a rare yet devastating complication after total joint replacement. The gold standard therapeutic options of PJI include infective tissues debridement and antibiotics treatment, with little attention to mechanical function, ending in an actual decoupling of infection and orthopedic treatment [53,54,55]. Two-stage revision surgery with antibiotic-loaded spacer implantation represents the standard of care for patients who develop chronic infection at the site of a total joint replacement [10]. Several papers have demonstrated the mechanical superiority of the articulated spacers with a more favorable outcome in terms of joint function and patient’s quality of life than static spacers, which are relegated to a few specific surgical indication [56]. Even though the success of the use of knee spacer has been reported to approach or to exceed 80% in many studies [57], a few recent studies have demonstrated that prosthesis removal for PJI is associated with significant morbidity and mortality and that many patients may not ultimately proceed to prosthesis reimplantation. In addition, failures may include persistent infection or mechanical issues such as fracture or dislocation of the spacer. Mechanical complications are widely correlated to the limited range of spacer sizes, which cannot cover all the anatomic variations such bone loss, joint line, malalignment, and joint function. Indeed, surgery performed using the “off-the-shelf” method aims to match the most appropriate implants for each patient and to obtain good functional results. Sometimes, in difficult knee revision scenarios, this could not be possible and the main effort of the surgeon is to avoid post-operative complications. Furthermore, when extensive or asymmetrical bone losses occur, standard spacers may not preserve the alignment, the stability, and the anatomical matching, thus causing failure of the procedure. In the following, a non-exhaustive list of drawbacks associated to the common use of ALPMMA spacers is reported, such as: (i) both femoral-tibial and femoral-patellar instability; (ii) the possibility of developing important contractures in flexion; (iii) frailty and subsequent breaking of the implant, especially with high doses of antibiotic; (iv) the difficulty of managing malalignments on the three planes of space (frontal, sagittal and transversal) and bone defects whether segmental or cavitary (affecting articular surfaces, metaphysis, and diaphysis); and (v) joint dislocation.

In this setting, the customization of the ALPMMA spacer aims to optimize bone implant fit and avoid spacer overhang or under-coverage, to improve ligament balancing by avoiding resection laxity, to improve mid-flexion stability and kinematics, to improve patellofemoral tracking by restoring the native femoral torsion, and to facilitate the restoration of the native limb alignment. In this paper, to better point out the role of the customized spacer on the frontal and axial malalignment secondary, due to a segmental bone loss, and on the restoration of the joint line, three different scenarios have been analyzed.

Placement of the joint line at natural level is paramount in revision knee surgery, and thus it influences the extension gap, the mid flexion stability, and the patellar height. Consequently, since bone loss symmetrically occur on medial and lateral femoral condyle, a distalization of femoral component should be performed, as shown in “Case N° 1”, in which, ideally, the surgeon should implant a femoral component, characterized by greater distal thickness both in the medial and lateral site. In our case series the proximalization of joint line occurred in the 16 % (3/19) of patients included in the study.

The type 2 medial epiphyseal defect according to AORI classification, described in “Case N° 2, generally, compromises the implant stability with a varus positioning of the femoral component. Modular augments are necessary to restore the mechanical femoral alignment. With the use of customizing implant, which presents a thicker medial distal femoral, the surgeon has the possibility of quickly applicating the implant, obtaining an effective reconstruction of areas of deficient bone and restoration of both level and stability of joint. Alignment defect restoration occurred in the 74 % (14/19) of patients included in the study.

Axial alignment of the femoral is also important for patella-femoral mechanics and the establishment of flexion-extension gap balance. A postero-lateral bone defect causes an internal rotation of the femoral component with consequently a flexion instability and alteration of patellar tracking. The correct position of the femoral component parallel to the surgical epicondylar axis as described in [58] needs the application of posterior femoral to augment as described in “Case N° 3”. Rotation defect restoration occurred in the 10 % (2/19) of patients included in the study.

## 5. Conclusions

The management of prosthetic joint infections (PJI) represents a major challenge for the orthopedic surgeon due to the diagnostic and therapeutic complexity and the exponential growth-rate of new cases of infection. An effective management of PJIs should include well-distinguished yet chained phases such as early diagnosis, surgical treatment and post-operative follow-up. This paper has been focused on treatment, as it significantly impacts on the patient’s personal autonomy and quality of life. In particular, the benefits offered by the customization process have been described. The tool used is a V3DPS, implemented in Matlab environment, in which a 3D model of the customizable ALPMMA spacer is available together with the 3D bone models of the patients under test. The main purpose of this process is to evaluate the cause-and-effect link between the geometric characteristic of the ALPMMA spacer and surgery failures. To meet the challenges involved in this, three common scenarios have been described, which are also useful in order to better highlight the advantages of using a customized knee spacer. It has been shown how the customized knee spacer, obtained by acting on its thicknesses, may restore the mechanical alignment and the joint line at the same time of infection treatment.

For these reasons, the proposed knee spacer could represent an all-in-one device that carries all the treatment solutions in one step.

This may be possible through customization in terms of size and the increase of the thickness symmetrically or asymmetrically according to bone loss pre-operatively evaluated. Lastly, patient specific devices could also reduce surgical time and, secondarily, both the peri-operative blood loss and complications rate. Further studies are needed to ulteriorly develop custom made spacers and to proceed in in vivo testing of the implant.

## 6. Patents

The 3D model of the ALPMMA spacer used in this paper is described in detail in the Italian Patent 2020. The patent number is 102020000005524 [59].

## Data Availability

The data presented in this study are available on request from the corresponding author.
